# Chemical Composition, Antioxidant, DNA Damage Protective, Cytotoxic and Antibacterial Activities of *Cyperus rotundus* Rhizomes Essential Oil against Foodborne Pathogens

**DOI:** 10.1038/srep45231

**Published:** 2017-03-24

**Authors:** Qing-Ping Hu, Xin-Ming Cao, Dong-Lin Hao, Liang-Liang Zhang

**Affiliations:** 1 School of Life Science, Shanxi Normal University, Linfen City, 041004, China; 2 School of Food Science, Shanxi Normal University, Linfen City, 041004, China

## Abstract

*Cyperus rotundus* L. (Cyperaceae) is a medicinal herb traditionally used to treat various clinical conditions at home. In this study, chemical composition of *Cyperus rotundus* rhizomes essential oil, and *in vitro* antioxidant, DNA damage protective and cytotoxic activities as well as antibacterial activity against foodborne pathogens were investigated. Results showed that α-cyperone (38.46%), cyperene (12.84%) and α-selinene (11.66%) were the major components of the essential oil. The essential oil had an excellent antioxidant activity, the protective effect against DNA damage, and cytotoxic effects on the human neuroblastoma SH-SY5Y cell, as well as antibacterial activity against several foodborne pathogens. These biological activities were dose-dependent, increasing with higher dosage in a certain concentration range. The antibacterial effects of essential oil were greater against Gram-positive bacteria as compared to Gram-negative bacteria, and the antibacterial effects were significantly influenced by incubation time and concentration. These results may provide biological evidence for the practical application of the *C. rotundus* rhizomes essential oil in food and pharmaceutical industries.

The excessive amounts of reactive oxygen species (ROS) and reactive species can lead to the peroxidation of lipids, glycation/oxidation/nitration of proteins, inactivation of enzymes, DNA damage and other alterations in the cellular organelles^1^,^2^. The oxidative DNA damage led to cell death and tissue damage, and is generally regarded as carcinogenic and actively participates in many pathological processes, including cancer and aging^3^. Supplementation of antioxidants can therefore prevent and protect the human body from oxidative stress related diseases^4^.

In recent years, food oxidation and food spoilage caused by microorganisms is one of the most important issues facing the food industry and consumers. Accompanied by growing consumer interest in natural food additives, the search for effective antioxidants and antibacterial agents from natural resources as an alternative to suppress food deterioration is now focused on edible plants, especially spices and herbs, due to their presenting fewer side effects than synthetic chemicals used in today’s foods^5^. There has been increasing realization in recent years that several plant derived essential oil may possess antioxidant, antimicrobial, anticancer and apoptosis inducing properties^6^,^7^. Therefore, the role of plant derived essential oil in chemoprevention of cancer has emerged as an interesting area of research.

*Cyperus rotundus* L. (Cyperaceae), is a smooth, erect and perennial weed and is widely distributed in tropical and warmer temperate regions worldwide. From the ancient time rhizomes and tubers of *C. rotundus* have long been used as an herbal remedy to treat stomach and disorders bowel and menstrual irregularities in several countries including China, India, Iran, and Japan^8^. Many researchers have reported the biological and pharmacological activities of *C. rotundus* extracts^2^,^8^,^9^,^10^,^11^,^12^,^13^. Chemical composition of the essential oil derived from *C. rotundus* rhizome had been widely studied^14^,^15^,^16^,^17^. Some studies also reported the antioxidant^6^,^18^, antibacterial activity^18^,^19^, insecticidal activity^20^ of the essential oil of *C. rotundus* rhizome, as well as antiradical and antimutagenic properties^21^,^22^. However, to the best of our knowledge, these informations are still limited.

The purpose of this study was to determine chemical composition of the essential oil of *C. rotundus* rhizome growing wild in China, and to further evaluate *in vitro* the antioxidant, oxidative DNA damage protective and cytotoxic effects as well as antibacterial activity of *C. rotundus* rhizome essential oil against several common foodborne pathogens for exploring its potential nutritional value and pharmacological activity in food and medicine.

## Results and Discussion

### Chemical compositions of the essential oil

The light yellow essential oil was obtained by hydrodistillation of dried *C. rotundus* rhizomes with a yield of 0.83% (v/w). The chemical compositions of essential oil were analyzed by GC-MS and the result was presented in Table 1. In total, 30 components were identified, representing 94.7% of the total amount. The α-cyperone (38.46%), cyperene (12.84%) and α-selinene (11.66%) was found to be the major components in the essential oil of *C. rotundus* rhizomes, followed by β-caryophyllene oxide (4.33%), (d)-limonene (3.62%), α-calacorene (3.14%), and γ-muurolene (3.13%), besides, other components (0.13–1.58%) were found to be lower content in the essential oil in the present study (Table 1). The profile obtained in the present study was very similar to the previous results reported by Liu *et al*. who found that α-cyperone (29.38%), cyperene (13.97%), caryophyllene oxide (6.71%) and β-selinene (6.47%) were the major compounds in the essential oil from Zhejiang province in China^20^. Aghassi *et al*. reported that cyperene (37.9%) and cyperotundone (11.2%) were the major components from oil of *C. rotundus* grown in Iran^16^. The rhizome oils of *C. rotundus* from Tunisia were reported to have cyperotundone (19.7%), cyperene (15.2%), mustakone (5.8%), caryophyllene oxide (2.6%), rotundene (3.6%) and eudesma 5-en-11-α-ol (2.6%)^18^. Lawal and Oyedeji reported that there was an obvious difference in components of essential oil between two different locations both in the Kwa-Zulu Natal Province of South Africa^15^. These differences in components and its content of essential oil from *C. rotundus* rhizome may be concerned in the chemotypes^15^, geographical region^16^,^23^, extracts methods^24^ and analysis methods^6^,^17^ of essential oil. According to previous research results that the volatile compounds of *C. rotundus* has been divided into four types four chemotypes (H-, K-, M-, O-types)^15^, the essential oil in the present study should belong to the M-type because it mainly contained α-cyperone, cyperene, caryophyllene oxide, and β-selinene.

### DPPH and ABTS radicals scavenging activity

The scavenging activity of essential oil from *C. rotundus* rhizomes on DPPH and ABTS radicals is shown in Table 2. The EC_50_ values of essential oil on DPPH and ABTS radicals were 75.0 and 36.1 μg/mL, respectively. The scavenging activity of essential oil on DPPH radicals was far lower than that of Trolox (EC_50_ was 13.1 μg/mL), however, its scavenging activity on ABTS radicals was significantly higher than Trolox (EC_50_ was 84.7 μg/mL). These differences in data between DPPH and ABTS assays were likely due to different experimental conditions. Similarly, essential oil showed a concentration-dependent scavenging of the DPPH and ABTS radicals at certain concentrations, indicating the antioxidant activity of essential oil may be mediated through direct trapping of the free radicals through transfers of hydrogen or electron^25^. In previous studies, Kilani *et al*.^6^ reported the antioxidant activity of essential oil determined by DPPH assay and superoxide anion-generating system, showing an inhibition percentage of 40% on DPPH radical at 0.1 mg/mL of *C. rotundus* essential oil; while Essaidi *et al*.^17^ also reported its antioxidant activity determined by DPPH assay and β-carotene bleaching test, indicating an inhibition percentage of 20% on DPPH radical and a dose-dependent effect. Unfortunately, they did not further determine the EC_50_ value of essential oil on DPPH radicals. In the present study, the scavenging rate was 57.6% at 0.1 mg/mL of *C. rotundus* essential oil on DPPH radicals (no shown). This difference in scavenging percentage could be explained by difference in the chemical composition and experimental conditions.

### Ferric reducing antioxidant power (FRAP)

**Figure 1:**
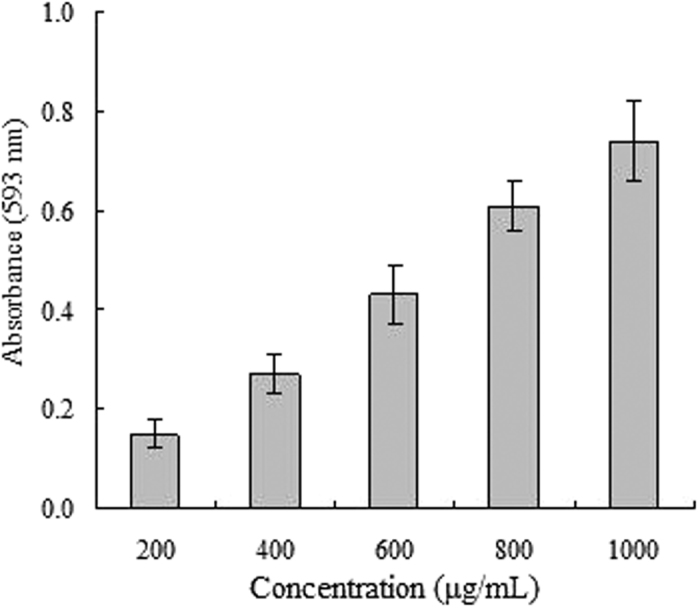
Reducing power of the essential oil from *C. rotundus* rhizomes.

The FRAP may serve as a significant indicator of the potential of antioxidant activity^26^. Figure 1 showed that the reducing power of essential oil from *C. rotundus* rhizomes was in a concentration-dependent manner and increased with the concentration, and the absorbance value significantly increased from 0.15 at 200 μg/mL to 0.74 at 1000 μg/mL. These results suggested that the essential oil from *C. rotundus* rhizomes could result in reducing Fe^3+^/ferricyanide complex to the ferrous form (Fe^2+^), and had a remarkable potency to donate electron to reactive free radicals, transforming them into more stable non-reactive species and terminating the free radical chain reaction.

### DNA damage protective effect

**Figure 2:**
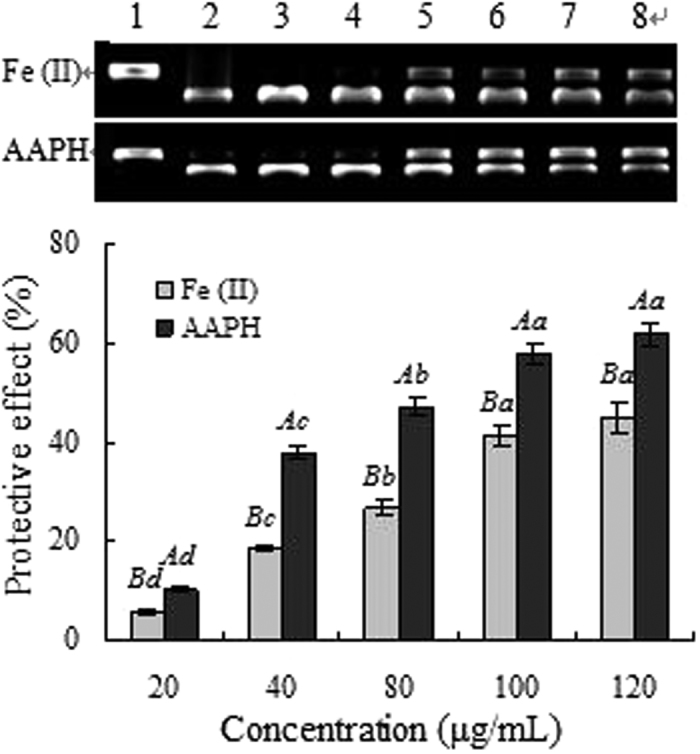
Protection effects of the essential oil from *C. rotundus* rhizomes on DNA oxidative damage induced by Fe^2+^ and AAPH. Lane 1, the native DNA; lane 2, the DNA treated with Fe^2+^/AAPH; lane3, the DNA treated with Fe^2+^/AAPH and solvent; lane 4–9, the DNA treated with the essential oil. Different lowercase letters indicate statistically significant differences between the means (*p* < 0.05) for essential oil of different concentration. Different capital letters indicate statistically significant differences between the means of DNA oxidative damage induced by Fe^2+^ and AAPH (*p* < 0.05) for essential oil at the same concentration.

The protection effects of the essential oil from *C. rotundus* rhizomes on DNA oxidative damage induced by Fe^2+^ and AAPH were evaluated and the results are shown in Fig. 2. Supercoiled plasmid DNA (Fig. 2, lane 1) was prone to oxidation by hydroxyl radicals or peroxyl radicals generated by AAPH, which resulted in the formation of open circular (Fig. 2, lane 2). From the gel analysis, similar results were found in protective effect assay of DNA from oxidative damage of Fe^2+^ and AAPH. The essential oil showed effective and concentration dependent reduction in the formation of nicked DNA and increased super coiled form of DNA. In concentration range from 20 to 100 μg/mL, the protective effects of the essential oil against DNA damage induced by Fe^2+^ and AAPH increased rapidly from 5.5% to 41.5% and from 10.4% to 58.0%, respectively. However, no significant change was found with the increase of concentration. These findings showed that the essential oil from *C. rotundus* rhizomes owned a higher potential to prevent DNA damage. Nonetheless, essential oil exhibited stronger protection effects of DNA oxidative damage induced by AAPH than Fe^2+^, which may come from different determination method^27^. The results of DNA oxidative damage induced by Fe^2+^ indicated that the essential oil might prevent the Fenton’s reaction, and or it probably quenched hydroxyl radicals by donating hydrogen-atom or electron^28^, while the other showed that the essential oil had the scavenging activity on peroxyl radicals generated by AAPH.

### Cytotoxicity of the essential oil

**Figure 3:**
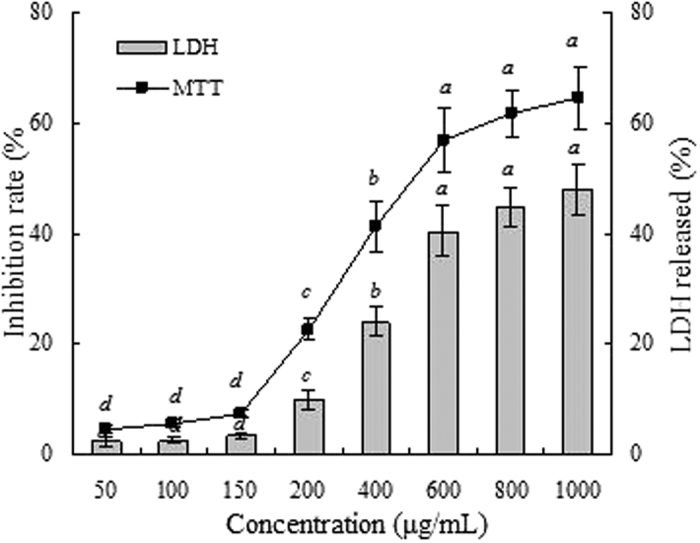
Cytotoxicity of the essential oil by MTT (bar) and LDH (line) assays.

The rhizome of *C. rotundus* has been reported to have the neuroprotective role^29^,^30^, anti-apoptotic and anxiolytic activity using SH-SY5Y human neurons^2^,^31^. In view of this, SH-SY5Y cells were selected to investigate the cytotoxicity of essential oil from *C. rotundus* rhizomes. The relationship between concentration of essential oil and their cytotoxic effect on SH-SY5Y cells was investigated by MTT and LDH release assays. Compared with untreated control cells, no significant change in the viability of SH-SY5Y cells was found when the concentration at 50–150 μg/mL. However, a significant decrease in cell viability and LDH leakage was observed above 150 μg/mL *C. rotundus* essential oil treatment (Fig. 3). Kilani *et al*. reported that the ethyl acetate extracts of *Cyperus rotundus* suppressed growth and proliferation of L1210 cells derived from murine lymphoblastic leukaemia by MTT assay^9^. Hemanth Kumar *et al*. also reported the cytotoxic activities of *C. rotundus* extracts in cell culture SH-SY5Y cells^2^. Kilani *et al*. suggested that *Cyperus rotundus* essential oil from Tunisia was very effective against L1210 leukaemia cells line by MTT assay^6^. These results supported the present study. The MTT assay determines cytotoxicity based on the mitochondrial damage of the cells, while LDH assay determines the cytotoxicity based on the plasma membrane damage of the cells. The present study indicated that *C. rotundus* essential oil had a higher cytotoxic effect on SH-SY5Y cells and the cytotoxic effect increased with a higher dosage in a certain concentration range. The cytotoxic activity of *C. rotundus* essential oil may be attributed to the presence of sesquiterpene hydrocarbons, oxygenated sesquiterpenes and monterpenens. Besides, the presence of antioxidants in the active *C. rotundus* may play some roles in reducing cell number because reactive oxygen radicals play an important role in carcinogenesis^32^.

### ZOI, MIC and MBC of the essential oil

The ZOI, MIC, and MBC values of the essential oil from *C. rotundus* rhizomes are presented in Table 3. The results showed that the essential oil had a satisfactory antibacterial activity on all of the tested Gram-positive and Gram-negative bacteria. The ZOI values of the essential oil were in the range of 10.5–24.4 mm for all tested bacterial strains, respectively. The MIC and MBC values for tested bacterial strains were in the range of 10–40 mg/mL and 20–40 mg/mL, respectively. Unfortunately, the MIC and MBC values of the essential oil for *E. coli* have not been gained when the concentration of essential oil reached the maximum in method system tested. Of these bacteria, the essential oil performed both a minimum MIC of 10 mg/mL and a minimum MBC of 20 mg/mL against *S. aureus* and *B. subtilis*, which indicated it was the most effective bacterial inhibitor and bactericide against *B. subtilis*. On the whole, the Gram positive bacteria were more sensitive than the Gram-negative ones to the essential oil from *C. rotundus* rhizomes (*p* < 0.05). To some extent, these results were consistent with previous studies on antibacterial activity of *C. rotundus* essential oils^17^,^33^, which was likely due to the significant differences in the outer layers of Gram-negative and Gram-positive bacteria. Resistance of Gram-negative bacteria against essential oils is attributed the presence of a hydrophilic outer membrane which possess hydrophilic polysaccharide chain as a barrier hydrophobic essential oil^34^,^35^,^36^.

### Fluorescence microscopy analysis

Based on the sensitivity of tested foodborne pathogens, one Gram-negative strain *S. aureus* and a Gram-negative strain *S. yphimurium* were selected as the model organisms to investigate the effect of essential oil from *C. rotundus* rhizomes on the viable counts of tested bacterial pathogen by fluorescence intensity changes of the bacteria.

**Figure 4:**
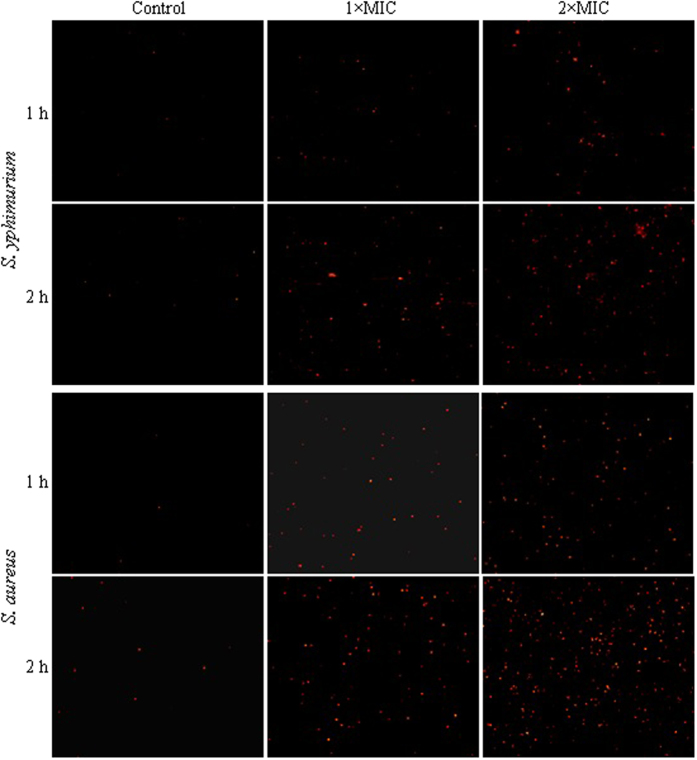
Fluorescence microscopy images of *S. yphimurium* and *S. aureus* treated with essential oil of different concentrations for 1 h and 2 h, respectively.

As observed in Fig. 4, the bacterial number and fluorescence intensity of the essential oil-treated *S. yphimurium* and *S. aureus* cells increased dramatically with incubation time and concentrations of essential oil. Taken together, these results confirmed the inhibiting capacity of essential oil on the growth rate of surviving *S. yphimurium* and *S. aureus*, and also suggested that incubation time and concentration presented significant inhibitory effects on the growth of tested bacterial strains.

## Conclusion

In summary, results from the present study indicated that the essential oil from *C. rotundus* rhizomes possessed an excellent antioxidant activity, as evidenced by *in vitro* DPPH, ABTS, and FRAP assays. The essential oil exhibited the protective effect against DNA oxidative damage induced by Fe^2+^ and AAPH, respectively. Moreover the essential oil also showed cytotoxic effects on the human neuroblastoma SH-SY5Y cell line and antibacterial activity against several foodborne pathogens. This study may provide biological evidence for the practical application of the *C. rotundus* rhizomes essential oil in food and pharmaceutical industries. However, further investigation of its activity *in vivo*, is necessary to elaborate and exploit this promise. Moreover, further studies should also include the molecular mechanism of the biological activity of the essential oil from *C. rotundus* rhizomes.

## Methods

### Plant materials

The rhizomes of *C. rotundus*, which were harvested in the region of Taian County of Shandong Province, China in 2015, were obtained as commercial products from the local market in March 2016. The moisture content, which was determined using a laboratory oven at 110 °C, was 11.4% for dried *C. rotundus* rhizomes.

### Chemicals and reagents

2, 4, 6-Tri (2-pyridyl)-s-triazine (TPTZ) were purchased from Fluka (Switzerland). 2,2′-azino-bis (3-ethylbenothiazoline-6-sulfonic acid) diammonium salts (ABTS), 2,2′-azobis (2-methylpropionamidine) dihydrochloride (AAPH), 3-(4,5-dimethyl-2-thiazolyl)- 2,5-diphenyl-2H-tetrazolium bromide (MTT), 2,2-Diphenyl-1-picrylhydrazyl (DPPH), the homologous series of *n*-hexane (C_8_-C_24_) and lactate dehydrogenase (LDH) activity assay kit were from Sigma (USA). Propidium iodide (PI) was from BD Biosciences. The pBR322 plasmid DNA was from Takara Bio Co. Ltd. (Dalian, China). Other chemicals used were all of analytical grade and obtained from China.

### Bacterial strains and culture

Three Gram-positive strains were *Staphylococcus aureus* (ATCC 25923), *Staphylococcus epidermidis* (ATCC 8799) and *Bacillus subtilis* (ATCC 6051). Three Gram-negative bacteria were *Escherichia coli* (ATCC 25922), *Salmonella typhimurium* (ATCC 19430), and *Shigella dysenteriae* (CMCC (B) 51252). Strains were provided by the School of Life Science, Shanxi Normal University, and cultured at 37 °C on nutrient agar and nutrient broth mediums.

### Essential oil extraction

The dried rhizomes of *C. rotundus* were ground with a micro plant grinding machine to a powder and then hydrodistilled for 6 h using a Clevenger-type apparatus. The oil was separated from water and dried over anhydrous sodium sulfate and stored in tightly closed dark vials at 4 °C until use.

### GC-MS analysis

The analysis of the essential oil was performed using a Hewlett-Packard 5890 II GC, equipped with a HP-5 MS capillary column (30 m × 0.25 mm; film thickness, 0.25 *μ*m) and a HP 5972 mass selective detector for the separation. The mass selective detector was operated in electron-impact ionization (EI) mode with a mass scan range from *m*/*z* 30 to 550 at 70 eV. Helium was the carrier gas at a flow rate of 1 mL/min. The initial temperature at 50 °C, held for 1 min, ramped at 5 °C/min to 280 °C and held for 1 min. Injector and MS transfer line temperatures were set at 230 and 300 °C, respectively. The oven temperature was programmed as in the GC-FID analysis. A sample of 1 *μ*L of 1% essential oil was injected manually using a 1:10 split ratio. Most components were identified by comparing their GC retention indices which were determined in relation to a homologous series of *n*-alkanes (C8-C24) under the same operating conditions, NIST mass spectral search program (version 2.0, National Institute of Standards and Technology), and mass spectra with publish data. Component relative percentages were calculated based on GC peak areas without using correction factors.

### DPPH assay

The scavenging rate and scavenging activity of the sample on DPPH radicals were determined according to the method as previously described^37^. The scavenging activity was expressed by EC_50_ value that is the effective concentration at which free radicals are scavenged by 50% and is obtained by interpolation from regression analysis.

### ABTS assay

The ABTS cation radical scavenging activity was determined according to the method as previously described^37^. The scavenging rate and EC_50_ value were calculated using the equation described for DPPH assay.

### Ferric reducing antioxidant power (FRAP) assay

The reducing ability was determined by using FRAP assay as previously described^37^. The absorption of the reaction mixture was measured at 593 nm after incubation for 30 min at 37 °C. Increased absorbance of the reaction mixture indicated increased reducing power.

### Protection of DNA oxidative damage induced by Fe^2+^

The ability of samples to protect supercoiled pBR322 plasmid DNA against Fe^2+^ and H_2_O_2_ was estimated with the DNA nicking assay as previously described^37^.

### Protection of DNA oxidative damage induced AAPH

The ability of samples to protect supercoiled pBR322 plasmid against AAPH was measured according to the method described by Zhang and Omaye with some modifications^38^. Intact pBR322 plasmid (0.5 μg) was mixed with various concentrations of samples and 2 μL of 25 mM AAPH in PBS (pH 7.4), and the mixture was incubated for 30 min at 37 °C. Then the samples were electrophoresed on 0.8% agarose gel containing 0.5 μg/mL ethidium bromide, photos of DNA bands were taken under gel image analysis system.

### Cytotoxicity activity

To assess the cytotoxic effects, the human neuroblastoma SH-SY5Y cell line was used in this study. Cells were seeded into 96 and 24-well plates in 1:1 mixture of DMEM/F-12 supplemented with 10% FBS, 2 mM L-glutamine, antibiotic and antimycotic solution in a humid atmosphere of 5% CO_2_ and 95% air at 37 °C. The media was changed on alternative days and once the confluency was reached, the cells were treated with sample at different concentrations. The MTT assay and lactate dehydrogenase (LDH) release assay was performed to determine the cell viability as described in a previous report^2^. Briefly, the SH-SY5Y cells were seeded in 96-well plates at a density of 1 × 10^4^ cells/well and grown for 36 h and then subjected with essential oil at concentrations ranging from 50 to 1000 μg/mL. After treatments, MTT (0.5 mg/mL) was added to each well and incubated for 2 h at 37 °C and the formed formazan crystals were dissolved in DMSO, and then the percentage of cell viability was calculated. For LDH release assay, the SH-SY5Y cells were plated at a density of 5 × 10^4^ cells/well in 24-well plates. After 24 h, the cells were treated with essential oil for 24 h and then lysed with, 10 μL of 2% Triton X-100. The cells were precipitated by centrifugation at 3000 rpm for 5 min at 4 °C. The supernatant (100 μL) was mixed with 900 μL of kit reaction mixture and the enzyme activity was measured in terms of intracellular LDH released into the medium at a wavelength of 340 nm.

### Antibacterial activity

The *in vitro* antibacterial activity of the tested sample was carried out by disc diffusion method. The inoculum suspension containing 1 × 10^7^ CFU/mL of bacteria was spread on nutrient agar medium uniformly. The sample was dissolved and then loaded on 6 mm sterile individual discs. The loaded discs were placed on the surface of medium and the diameter of zone of inhibition (ZOI) was measured after 24 h of incubation at 37 °C. Minimum inhibitory concentration (MIC) and minimum bactericide concentration (MBC) of sample were determined according to the method as previously described^39^.

### Fluorescence microscopy analysis

Logarithmic phase bacteria were collected by centrifugation at 6000 × g for 5 min, washed three times, and resuspended in PBS (pH 7.4). Tested bacteria were treated with different concentrations of samples and incubated at 37 °C. After 1 h and 2 h, cells containing approximately 1 × 10^8^ CFU/mL were harvested by centrifugation at 6000 × g for 5 min and stained for 15 min with the equal volume of 1 mg/mL PI in the dark at room temperature. Ten microliters of the stained bacterial suspensions were dropped onto glass slides and covered with coverslips, and images were captured by a fluorescence microscope (Leica, DMi8).

### Statistical analysis

One-way analysis of variance (ANOVA) and Duncan’s multiple range tests were carried out to determine significant differences (*p* < 0.05) between the means by Data Processing System (DPS, version 7.05) and EXCEL program.

## Additional Information

**How to cite this article:** Hu, Q.-P. *et al*. Chemical Composition, Antioxidant, DNA Damage Protective, Cytotoxic and Antibacterial Activities of *Cyperus rotundus* Rhizomes Essential Oil against Foodborne Pathogens. *Sci. Rep.* **7**, 45231; doi: 10.1038/srep45231 (2017).

**Publisher's note:** Springer Nature remains neutral with regard to jurisdictional claims in published maps and institutional affiliations.

## Figures and Tables

**Table 1:** Chemical composition of essential oil from *C. rotundus* rhizomes.

Compounds	Percentage (%)					
	present result	reference 16	reference 19	reference 15		reference 18
				sample A	sample B	
α-Pinene	1.10	2.2	0.82	3.0	10.8	—
Camphene	0.35	—	—	—	1.5	—
β-Thujene	—	—	1.95	—	—	—
β-Pinene	0.34	3.9	0.67	5.3	11.3	—
ρ-Cymene	1.45	—	0.86	1.7	0.6	—
(d)-Limonene	3.62	—	3.31	2.0	5.7	—
1,8-Cineole	1.50	—	0.67	—	—	—
Linalool	1.05	—	0.89	—	—	—
Pinocarveol	0.83	—	2.15	—	—	—
Terpinenol	1.46	—	—	0.6	—	—
*trans*-pinocarveol	—	—	—	4.0	4.0	—
pinocarvone	—	—	—	2.2	0.4	—
terpinen-4-ol	—	—	—	0.9	1.0	—
*allo*-aromadendrene	—	—	—	1.2	0.8	—
Terpineol	—	—	0.65	—		—
p-Cymen-8-ol	0.52	—	1.67	—		—
Myrtenol	1.54	—	2.34	7.9	7.1	—
Verbenone	0.43	—	0.74	0.6	1.1	—
*trans*-Carveol	1.08	—	1.23	—	0.4	—
Carvone	0.51	—	0.94	—	0.2	—
Bornyl acetate	0.74	0.2	0.35	—	—	—
*Trans*-anethole	1.58	—	1.65	—	—	—
α-Copaene	1.24	—	1.44	—	0.5	0.6
β-Elemene	0.35	—	0.67	0.8	0.5	0.4
Cyperene	12.84	37.9	13.97	1.6	2.6	15.2
Gurjunene	0.14	—	1.94	—	0.3	—
γ-Muurolene	3.13	—	2.29	—	—	0.8
α-Selinene	11.66	1.3	—	2.7	6.6	0.1
β-Selinene	—	—	6.47	5.1	4.6	0.2
α-Muurolene	0.26	—	0.34	—	—	0.4
γ-Cadinene	0.13	0.1	0.71	—	—	0.3
Isolongifolen-5-one	—	—	1.24	—	—	
α-Cadinene	—	—	0.11	—	—	0.4
α-Calacorene	3.14	—	1.12	—	—	0.9
Spathulenol	0.45	—	4.17	—	—	1.4
β-Caryophyllene oxide	4.33	—	6.71	0.8	0.6	2.6
α-Cyperone	38.46	4.3	29.38	11.0	7.9	—
Aristolone	0.22	—	2.01	2.5	1.6	—
Nootkanone	0.25	—	1.24	—	0.2	3.8
α-Cubebene	—	3.7	—	—	—	0.4
Caryophyllane-2-6-β-oxide	—	0.2	—	5.4	2.6	—
α-Humulene	—	0.2	—	0.4	0.2	0.4
Vulgarol B	—	—	—	3.8	1.8	—
T-Calamenene	—	1.9	—	—	—	—
Caryophyllenol	—	—	—	4.8	0.9	—
Isorotundene	—	9.5	—	—	—	3.6
Isocyperol	—	2.1	—	—	—	—
Cyperol	—	6.4	—	—	—	—
T-Cadinol	—	2.9	—	—	—	—
Muurolol	—	3.0	—	—	—	1.8
α-Cadinol	—	1.8	—	—	—	1.9
Mustakone	—	3.7	—	—	—	5.8
Cyperotundone	—	11.2	—	—	—	19.7
Calamanene	—	—	—	—	—	0.7
Nardol	—	—	—	—	—	1.0
Humulene epoxide	—	—	—	—	—	1.5
Eudesma 5-en-11-α-ol	—	—	—	—	—	2.6
*epi*-Cubenol	—	—	—	—	—	0.8
Intermediol	—	—	—	—	—	0.7

**Table 2:** DPPH and ABTS radicals scavenging capacity of essential oil from *C. rotundus* rhizomes.

	Scavenging capacity	
	Regression equation	EC_50_ (μg/mL)
DPPH	y = 0.0024x + 0.3200 R^2^ = 0.9898	75.0 ± 4.1
ABTS	y = 0.1758ln(x) − 0.1305 R^2^ = 0.9977	36.1 ± 2.4

Values represent means of three independent replicates ± SD. R^2^ refers to the regression coefficients.

**Table 3:** ZOI, MIC, and MBC of essential oil from *C. rotundus* rhizomes.

Bacterial strains	ZOI (mm)^a^	MIC (mg/mL)	MBC (mg/mL)
**Gram**-**positive**			
*S. aureus*	22.3 ± 1.9 a	10	20
*S. epidermidis*	21.5 ± 1.2 a	20	40
*B. subtilis*	24.4 ± 1.5 a	10	20
**Gram**-**negative**			
*S. yphimurium*	16.3 ± 1.1 b	40	40
*E. coli*	10.5 ± 0.8 c	>40	NT^b^
*S. ysenteriae*	15.4 ± 1.3 b	40	40

^a^Values represent means of three independent replicates ± SD.

^b^NT, not tested. Different letters within a column indicate statistically significant differences between the means (*p* < 0.05) for ZOI.
